# Role of PKCδ in Enhanced Expression of Gqα/PLCβ1 Proteins and VSMC Hypertrophy in Spontaneously Hypertensive Rats

**DOI:** 10.1371/journal.pone.0157955

**Published:** 2016-07-05

**Authors:** Mohammed Emehdi Atef, Madhu B. Anand-Srivastava

**Affiliations:** Department of Molecular and Integrative Physiology, Faculty of Medicine, University of Montreal, Montreal, Quebec, Canada; Universidade Federal do Rio de Janeiro, BRAZIL

## Abstract

Gqα signaling has been implicated in cardiac hypertrophy. In addition, angiotensin II (Ang II) was also shown to induce its hypertrophic effect through Gqα and PKCδ activation. We recently showed the role of enhanced expression of Gqα/PLCβ1 proteins in vascular smooth muscle cell (VSMC) hypertrophy, however, the role of PKCδ in VSMC hypertrophy in animal model is still lacking. The present study was therefore undertaken to examine the role of PKCδ and the associated signaling mechanisms in VSMC hypertrophy using 16-week-old spontaneously hypertensive rats (SHR). VSMC from 16-week-old SHR exhibited enhanced phosphorylation of PKCδ-Tyr^311^ and increased protein synthesis, marker of hypertrophy, as compared to WKY rats which was attenuated by rottlerin, an inhibitor of PKCδ. In addition, knocking down of PKCδ by PKCδ-siRNA also attenuated enhanced protein synthesis in VSMC from SHR. Furthermore, rottlerin attenuated the increased production of superoxide anion, NAD(P)H oxidase activity, increased expression of Gqα, phospholipase C (PLC)β1, insulin like growth factor-1 receptor (IGF-1R) and epidermal growth factor receptor (EGFR) proteins in VSMC from SHR. In addition, the enhanced phosphorylation of c-Src, PKCδ-Tyr^311^, IGF-1R, EGFR and ERK1/2 exhibited by VSMC from SHR was also attenuated by rottlerin. These results suggest that VSMC from SHR exhibit enhanced activity of PKCδ and that PKCδ is the upstream molecule of reactive oxygen species (ROS) and contributes to the enhanced expression of Gqα and PLCβ1 proteins and resultant VSMC hypertrophy involving c-Src, growth factor receptor transactivation and MAP kinase signaling.

## Introduction

Essential hypertension is associated with vascular remodeling characterized by enhanced media to lumen ratio in arteries [1] and is due to increased vascular smooth muscle cell (VSMC) proliferation and hypertrophy. Guanine nucleotide regulatory proteins (G-proteins) and receptor tyrosine kinases (RTKs) play a major role in the regulation of vascular remodeling and aberration in the expression and/or activity of these molecules contribute to vascular remodeling [2–6]. The Gqα, a heterotrimeric G protein, and phospholipase C (PLC) β regulate phosphatidyl inositol (PI) turnover activated by many GPCR agonists such as angiotensin II (Ang II), endothelin-1 (ET-1) and thrombin, and play an important role in mediating the prohypertrophic response by initiating other signaling mechanisms including RTKs transactivation and MAP kinase activation [7–10]. The levels of Ang II and ET-1 are enhanced in VSMC from spontaneously hypertensive rats (SHR) [11,12] and promote VSMC hypertrophy [2] and proliferation [13] in an autocrine and paracrine way.

Postreceptor signaling pathways activated by growth promoting substances involve activation of protein kinase C (PKC) through 1,2-diacylglycerol (DAG) production and/or intracellular calcium [8,14]. PKC is an intracellular serine/threonine protein kinase family of at least 12 isotypes subdivided into three classes, conventional PKCs (cPKCs), novel PKCs (nPKCs) and atypical PKC (aPKCs), which have distinct functions. PKCs isozymes expression pattern vary according to cell type. PKCδ, PKCα and PKCζ are the most abundant isozymes in VSMC [15,16]. The role of PKC isoforms in vascular hypertrophy is still insufficiently characterized and may vary according to cell type [17,18]. These intracellular serine/threonine kinases are rapidly activated and are implicated in the regulation of cell proliferation [19] and growth [20] and likely play an important role in mediating vascular remodeling. PKCδ is one of nPKCs isoforms that do not require Ca^2+^ but is activated by DAG [21–24]. During the last decade, PKCδ associated with tyrosine (Tyr)^311^ phosphorylation has emerged as a potential mediator in response to many stimuli including Ang II and thrombin [7,25]. Furthermore, the involvement of PKCδ in growth factor activation such as EGFR [8,9,26,27] and IGF-1R [28] has also been reported. However, the role of PKCδ in mediating vascular remodeling in essential hypertension and its possible cross-talk with other signaling mechanism implicated in this process has not yet been well characterized. We recently demonstrated the role of endogenous Ang II and ET-1 in enhanced expression of Gqα and PLCβ1 proteins and VSMC hypertrophy in spontaneously hypertensive rats through the activation of MAPK signaling [2]. We also showed that enhanced oxidative stress exhibited by VSMC from SHR through c-Src and growth factor receptor activation increases MAP kinase signaling and enhances the expression of Gqα and PLCβ1 proteins and results in VSMC hypertrophy [29]. However, the role of PKCδ in mediating vascular remodeling in essential hypertension has not yet been well characterized. The present study is therefore undertaken to examine if VSMC from SHR exhibit enhanced activation of PKCδ and its implication in VSMC hypertrophy and to further explore the signaling mechanism responsible for this process.

We showed that the enhanced activation of PKCδ in VSMC from SHR increases oxidative stress, c-Src and growth factor receptor transactivation that through MAP kinase signaling increases the expression of Gqα and PLCβ1 proteins and results in VSMC hypertrophy.

## Materials and Methods

### Materials

Rottlerin and lucigenin were purchased from Sigma-Aldrich Chemical (St-Louis, Missouri, USA). Leucine, L-[4,5-3H(N)] was purchased from Perkin Elmer (Boston, MA). Monoclonal Gqα antibody (10), monoclonal PLC-β1 antibody (D-8), monoclonal (phospho)-ERK1/2 (phosphospecific-tyrosine-204) antibody, polyclonal (phospho)-PKCδ (phosphospecific-tyrosine-311)-R antibody, monoclonal PKCδ, polyclonal ERK1/2 antibody (C-14), and Western blotting reagents were from St Cruz biotech (Santa Cruz, CA, USA). Polyclonal (phospho)-EGFR antibody (phosphospecific-tyrosine-1173) was from Calbiochem. Polyclonal EGFR, IGF-1R, (phospho)-c-Src (phosphospecific-tyrosine-419) and (phospho)-IGF-1R (phosphospecific-tyrosine-1165/1166) antibodies were from St Cruz biotech. Monoclonal anti-β-actin antibody (A5441) and all other chemicals used in these experiments were purchased from Sigma-Aldrich (St. Louis, MO). Male spontaneously hypertensive rats (SHR) and age-matched Wistar Kyoto rats (WKY) were purchased from Charles River (St-Constant, Quebec, Canada).

### Animals, cell culture and incubation

16-week-old spontaneously hypertensive rats (SHR) and age-matched Wistar Kyoto (WKY) rats were euthanized by decapitation. The aorta were dissected out and VSMC were cultured as described previously [30]. As reported earlier [31], these cells were found to contain high levels of smooth muscle-specific actin. Cells were plated in 75-cm^2^ flasks and incubated at 37°C in 95% air-5% CO_2_ humidified atmosphere in Dulbecco’s modified Eagle’s medium (DMEM) (with glucose, L-glutamine, and sodium bicarbonate) containing 1% antibiotics (containing penicillin, streptomycin, and amphotrecin B) and 10% heat-inactivated fetal bovine serum (FBS). Cells were passaged upon reaching confluence with 0.5% trypsin and utilized between passages 2 and 6. Confluent cells were starved by incubation for 24 hours in DMEM without FBS at 37°C to have cell quiescence. For the receptor antagonist studies, VSMC from SHR and WKY were incubated for 16 hours in the absence or presence of variou concentrations of rottlerin (1 μM to 10 μM) dissolved in 1% dimethyl sulfoxide (DMSO). After incubation, the cells were washed twice with ice-cold phosphate-buffered saline (PBS) and lysed in a 200 μl buffer containing 25 mM Tris·HCl (pH 7.5), 25 mM NaCl, 1 mM sodium orthovanadate, 10 mM sodium fluoride, 10 mM sodium pyrophosphate, 2 mM EDTA, 1 mM phenylmethylsulfonyl fluoride, 10 μg/ml aprotinin, 1% Triton X-100, 0.1% sodium dodecyl sulfate, and 0.5 μg/ml leupeptin on ice. The cell lysates were centrifuged at 12,000 *g* for 15 min at 4°C, and the supernatants were used for Western blot analysis. Cell viability was checked by the trypan blue exclusion technique and indicated that >90~95% cells were viable. All the animal procedures used in the present study were approved by the Comité de Déontologie de l'Expérimentation sur les Animaux (CDEA) of the University of Montreal (#99050). The investigation conforms to the 'Guide for the Care and Use of Laboratory.

### Western blotting

The levels of protein expression and phosphorylation were determined by Western blotting as described previously [2]. After SDS-PAGE, separated proteins were transferred to a nitrocellulose membrane with a semi-dry transblot apparatus at 15 V for 45 min (Gqα, ERK1/2/p-ERK1/2) or a liquid transfer apparatus at 100 V for 1 h (PLCβ1, EGFR, pEGFR, IGFR, pIGFR, PKCδ, pPKCδ, c-Src and p-Src). Membranes were blocked with 5% dry milk and incubated overnight with specific antibodies. β-actin was used as loading controls. The antibody-antigen complexes were detected by incubating the membranes with horseradish peroxidase-conjugated antibodies for 1 h at room temperature. The blots were washed three times with PBS before reaction with enhanced chemiluminescence (ECL). Quantitative analysis of the proteins was performed by densitometric scanning of the autoradiographs using the enhanced laser densitometer LKB Ultroscan XL and quantified using the gel-scan XL evaluation software (version 2.1) from Pharmacia (Baie d’Urfé, Québec, Canada).

### Determination of protein synthesis

VSMC from 16 week-old SHR and age-matched WKY were grown to confluence in 12-well culture plates. Protein synthesis (cell hypertrophy) was evaluated by [^3^H]-leucine incorporation into cells as described previously [32]. Confluent cells were serum deprived for 24 hours to induce cell quiescence and were incubated in the absence or presence of rottlerin (1 to 10 μM) for 24 h. [^3^H]-leucine (2μCi per well) was also added at the same time as rottlerin. For RNA interference studies, the cells were incubated in the absence or presence of siRNA against PKCδ. [^3^H]-leucine was added and further incubated for 24 h before the cells were harvested. The cells were rinsed twice with ice-cold 1X PBS and incubated with 5% TCA for 1 h at 4°C. After being washed twice with ice-cold 1X PBS, the cells were incubated with 0.4 N sodium hydroxide solution for 30 min at room temperature, and radioactivity was determined by liquid scintillation counter and adjusted by protein concentration.

### Transfection of VSMC with siRNA

For siRNA transfection efficiency, the manufacturer’s protocol was followed. Briefly, VSMC were seeded in a 12 well plate or petri dishes and cultured in antibiotic free normal growth medium supplemented with 10% FBS until the cells were 60% confluent (~48 hours). On the day of transfection, cells were washed with transfection medium (sc-36868) and incubated with 1ml of transfection reagent (sc-29528) containing 80 pmoles of either scrambled siRNA (sc-37007) or siRNA specific for PKCδ oligonucleotides for 12 hours. The medium was replaced with normal DMEM (containing 10% FBS and 1% antibiotics) for an additional 24 hours (90% confluence).

### Determination of superoxide anion and NADPH oxidase activity

The production of basal superoxide anion (O_2_^-^) was measured using the lucigenin-enhanced chemiluminescence method at a concentration of 5 μM as described previously [33]After 24 hours of starvation, the VSMC were washed with oxygenated Krebs-Hepes buffer (NaCl 50 mM, KCl 2.3 mM, MgSO4 1.2 mM, K2HPO4 0.5 mM, NaHCO3 25 mM, glucose 5.5 mM, EDTA 63.4 μM) and placed in scintillation vials containing lucigenin solution. The emitted luminescence was measured with a liquid scintillation counter (Wallac 1409; Perkin Elmer Life Science, Saint-Laurent, Canada) for 5 min. The NAD (P) H oxidase activity was determined by adding NADH (100 μM). The values were adjusted to the total weight of proteins for each simple.

### Statistical analysis

Results are expressed as means ± SEM. Comparisons between groups were made with one-way analysis of variance (ANOVA) followed by Bonferroni's post-hoc test using GraphPad Prism5. A difference between groups was significant at P < 0.05.

## Results

### VSMC from SHR exhibit enhanced expression of PKCδ phosphorylation at Tyr^311^

**Fig 1:**
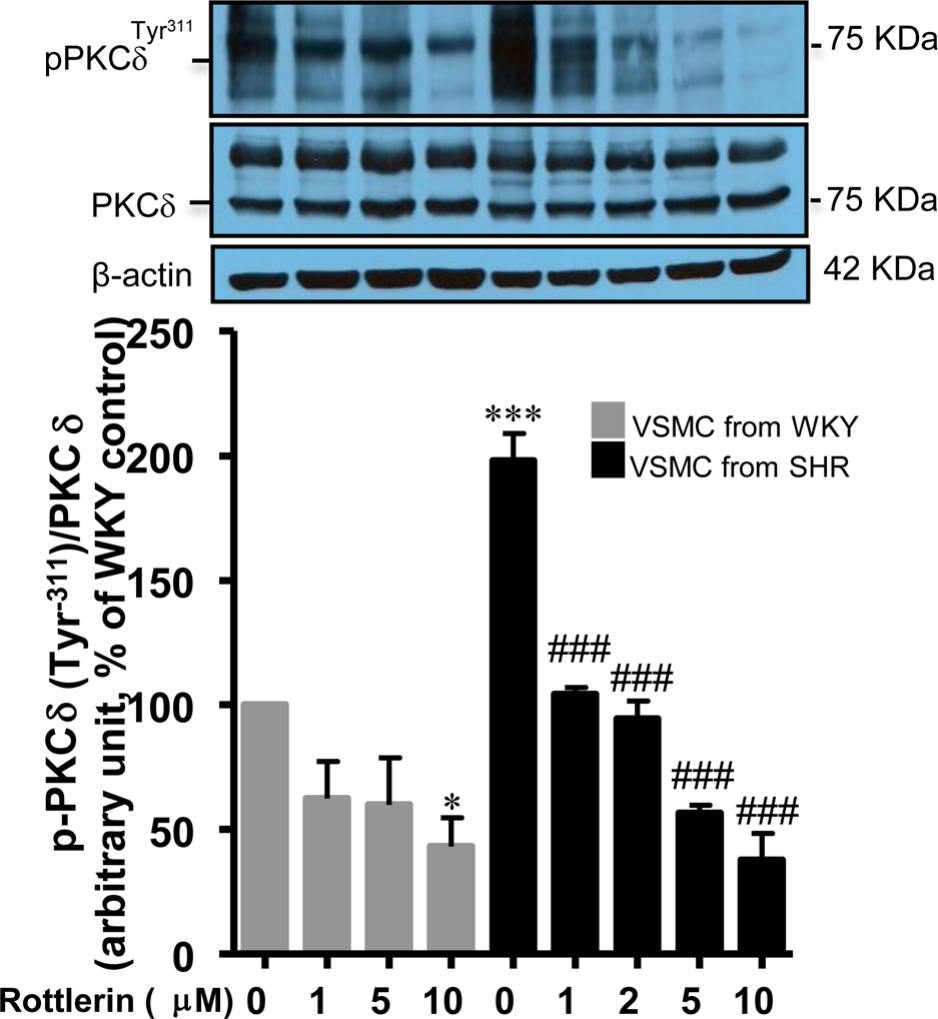
Effect of rottlerin on PKCδ phosphorylation at Tyr^311^ in VSMC from 16-weeks old SHR and age-matched WKY rats. VSMC from 16 week-old SHR and age matched WKY rats were incubated with different concentrations of rottlerin for 16 hours. The cell lysates were prepared and subjected to Western blotting using specific antibodies against PKCδ and (phospho)-PKCδ^311^ as described in Materials and Methods. PKCδ phosphorylation level was normalized by total PKCδ and the β-actin was used as a loading control. The proteins were quantified by densitometric scanning. The results are expressed as percentage of control, taken as 100%. Values are means ± SEM of 5 separate experiments using different cell cultures. *P < 0.05, ***P < 0.001 vs. WKY rats; ### P < 0.001 vs. SHR.

Several vasoactive peptides such as Ang II and ET-1 activate PKCδ [14, 34]. PKCδ activation associated with phosphorylation at Tyr^311^ has been shown to mediate Ang II–induced VSMC hypertrophy [7]. Since VSMC from SHR exhibit enhanced levels of endogenous Ang II [11] and enhanced protein synthesis [2], it was desirable to investigate if VSMC hypertrophy in SHR is attributed to the overexpression of PKCδ phosphorylated at Tyr^311^. To test this, we determined the levels of PKCδ phosphorylated at Tyr^311^ in VSMC from SHR. The results shown in Fig 1 indicate that the level of PKCδ protein phosphorylated at Tyr^311^ was significantly enhanced by about 100% in VSMC from SHR as compared to VSMC from WKY rats whereas the expression of PKCδ protein was not altered. Rottlerin, an inhibitor of PKCδ, attenuated significantly the enhanced Tyr^311^ phosphorylation in a concentration-dependant manner and at 10 μM, it was completely abolished. In addition, the phosphorylation level of PKCδ at Tyr^311^ in VSMC from WKY rats was also attanuated by rottlerin by about 60%.

### Role of PKCδ in VSMC hypertrophy in SHR

**Fig 2:**
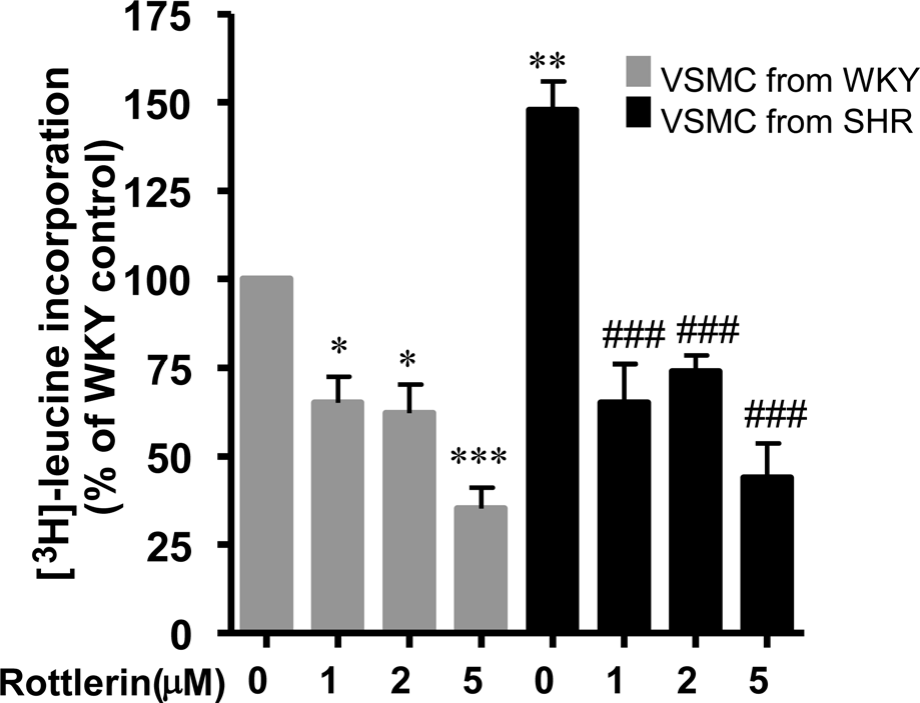
Effect of rottlerin on protein synthesis in vascular smooth muscle cells (VSMC) from 16-weeks old SHR and age-matched WKY rats. VSMC from 16 week-old SHR and age matched WKY rats were incubated with different concentration of rottlerin (from 1μM to 5μM). Protein synthesis was determined by [^3^H]-leucine incorporation as described in Materials and Methods. The results are expressed as a percentage of control, taken as 100%. Values are means ± SEM of 4 separate experiments using different cell cultures. *P < 0.05, **P < 0.01, ***P < 0.001 vs. WKY rats; ### P < 0.001 vs. SH.

VSMC from SHR have been shown to exhibit hypertrophy (increased protein synthesis) [2]. To investigate the role of PKCδ in enhanced protein synthesis in VSMC from SHR, the effect of rottlerin was tested on protein synthesis in VSMC from SHR and age- matched WKY rats and the results are shown in Fig 2. As reported earlier [2, 35], protein synthesis was significantly augmented in VSMC from SHR by about 50% as compared to WKY rats and rottlerin attenuated the enhanced protein synthesis at all the concentrations used in this study. At 5μM, the inhibition was about 90%. In addition, the protein synthesis in VSMC from WKY rats was also inhibited by rottlerin treatment.

**Fig 3:**
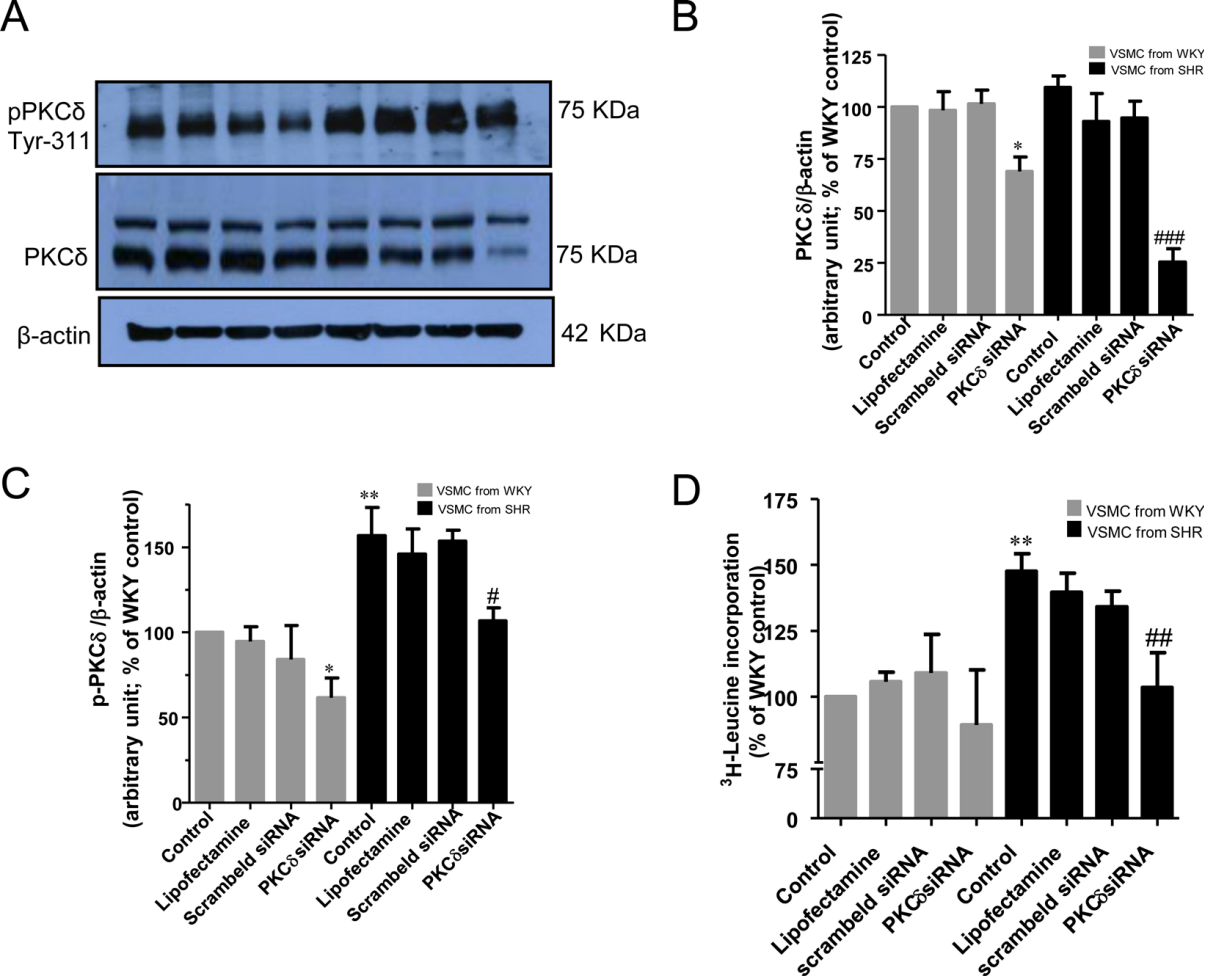
Effect of the knockdown of PKCδ on protein synthesis in vascular smooth muscle cells (VSMC) from 16-week-old SHR and age-matched WKY rats. VSMC from 16 week-old SHR and age matched WKY rats were incubated in the absence or presence of PKCδsiRNA for 16 hours as described in Materials and Methods. The cell lysates were prepared and subjected to Western blotting using specific antibodies against PKCδ and (phospho) PKCδ^311^ (A). PKCδ phosphorylation level was normalized by total PKCδ (C) and the levels of PKCδ were normalized by β-actin used as a loading control (B). The proteins were quantified by densitometric scanning (B, C) and the protein synthesis was determined by [^3^H]-leucine incorporation (D) as described in Materials and Methods. The results are expressed as percentage of control, taken as 100%. Values are means ± SEM of 5 separate experiments using different cell cultures. *P < 0.05, **P < 0.01 vs. WKY rats; # P < 0.05, ## P < 0.01, ### P < 0.001 vs. SHR.

To further investigate the implication of PKCδ in increased protein synthesis in VSMC from SHR, we used the siRNA approach to knockdown the PKCδ and examined the effect of knockdown of PKCδ on protein synthesis in VSMC from SHR and WKY rats. Results shown in Fig 3, indicate that the treatment of VSMC with siRNA of PKCδ that attenuated the expression of PKCδ by about 80% **(**A, B) also inhibited the enhanced protein synthesis by about 70% (D) in VSMC from SHR. However, a small but significant inhibition (≈ 25%) of the protein expression of PKCδ was observed in VSMC from WKY rats **(**A, B) whereas the protein synthesis was not significantly inhibited in these cells (D). In addition, PKCδ knockdown also attenuated slightly but significantly the PKCδ phosphorylation at Tyr^311^ in VSMC from SHR and age- matched WKY rats (A, C).

### Role of PKCδ in enhanced expression of Gqα and PLCβ1 proteins in VSMC from SHR

**Fig 4:**
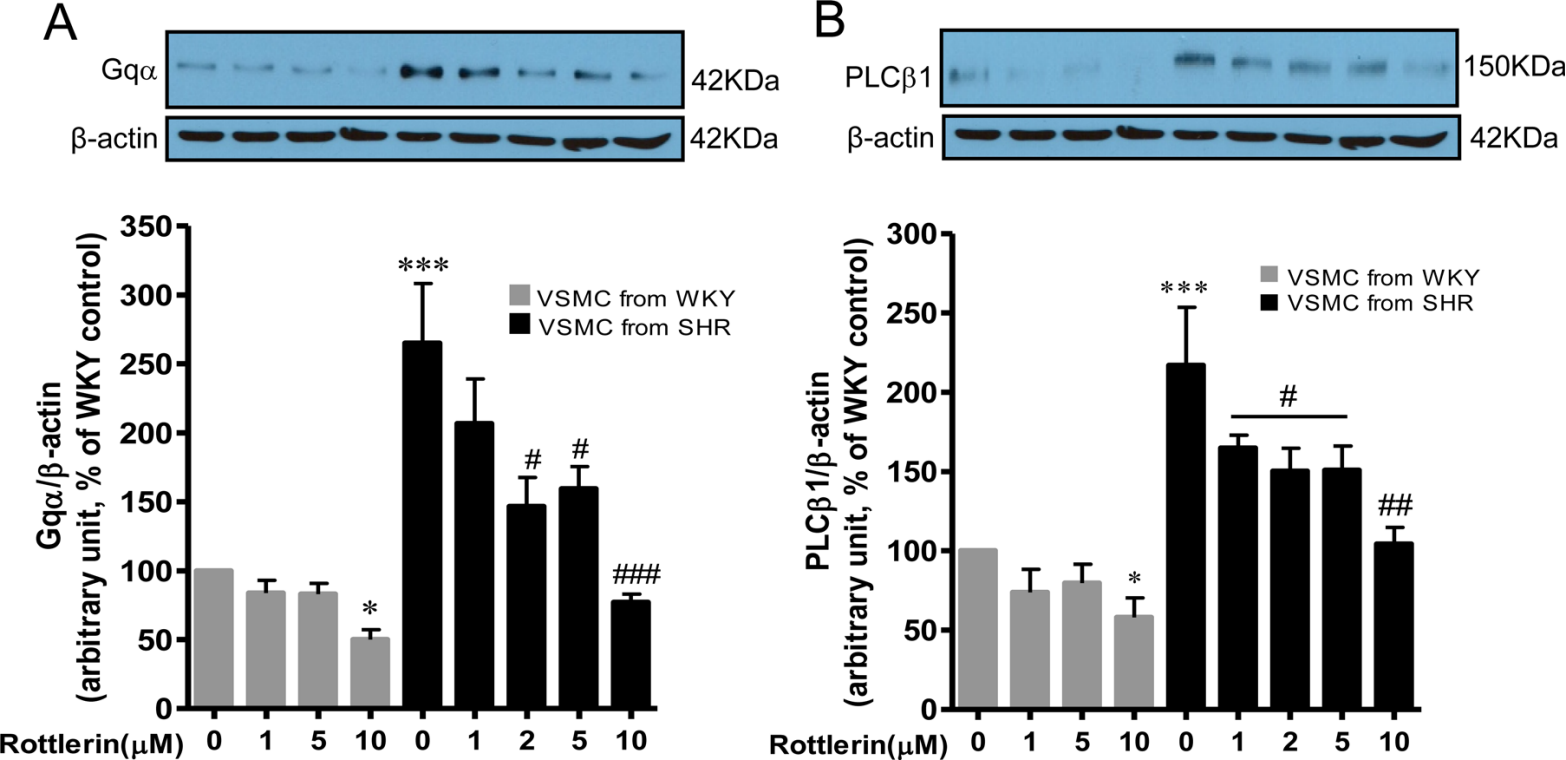
Effect of rottlerin on enhanced expression of Gqα/PLCβ1 in vascular smooth muscle cells (VSMC) from 16-weeks old SHR and age-matched WKY rats. VSMC from 16-weeks old SHR and age matched WKY rats were incubated with different concentrations of rottlerin. The cell lysates were prepared and subjected to Western blotting using specific antibodies against Gqα and PLCβ1 (A, B upper panels) as described in Materials and Methods. The β-actin was used as a loading control. The proteins were quantified by densitometric scanning (A, B lower panels). The results are expressed as percentage of control, taken as 100%. Values are means ± SEM of 4 separate experiments using different cell cultures. *P < 0.05, ***P < 0.001 vs. WKY rats; # P < 0.05, ## P < 0.01, ### P < 0.001 vs. SHR.

We have earlier shown the implication of enhanced expression of Gqα and PLCβ1 in enhanced protein synthesis in VSMC from 16 week-old SHR [2]. Since PKCδ is implicated in enhanced protein synthesis, it was of interest to examine if PKCδ contributes to the enhanced expression of Gqα and PLCβ1 in VSMC from SHR. To test this, the effect of rottlerin on the expression of Gqα and PLCβ1 was investigated in VSMC from SHR and WKY rats and the results are shown in Fig 4. As reported earlier [2], the expression of Gqα (A) and PLCβ1 (B) was significantly augmented by about 165 and 115% respectively in VSMC from SHR as compared to WKY rats and rottlerin significantly inhibited the enhanced expression of Gqα and PLCβ1 proteins and at 10 μM, it was completely attenuated to WKY control level.

### Implication of PKCδ in enhanced production of superoxide anion and NADPH oxidase activity in VSMC from SHR

**Fig 5:**
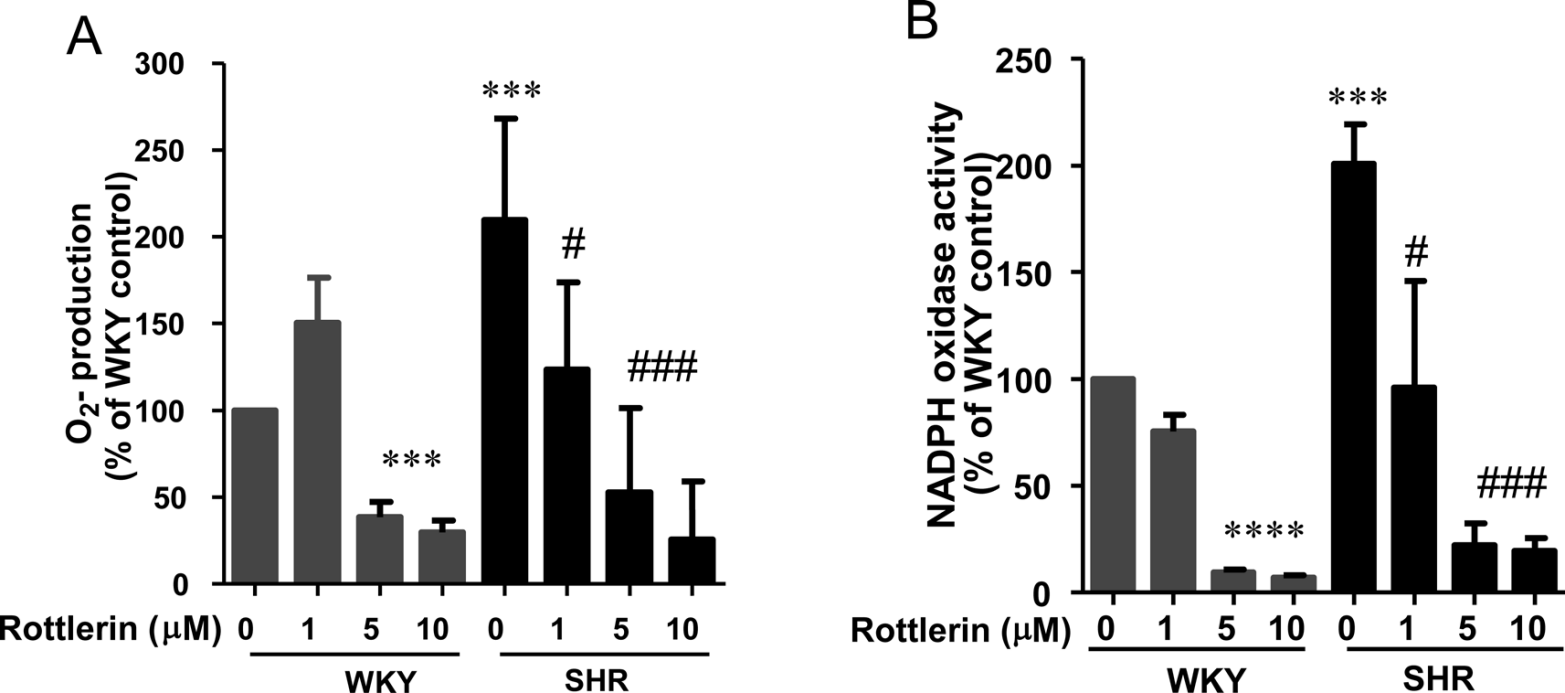
Effect of rottlerin on NADPH activity and ROS production in vascular smooth muscle cells (VSMC) from 16-weeks old SHR and age-matched WKY rats. VSMC from 16 week-old SHR and age matched WKY rats were incubated in the absence or presence of different concentration of rottlerin (from 1μM to 10 μM) for 16 hours, and O.^-^2 production (A) and NADPH activity (B) were determined as described in Materials and Methods. The results are presented as means ± SEM of 4 separate experiments using different cell cultures. ***P < 0.001 vs. WKY rats; # P < 0.05, ### P < 0.001 vs. SHR.

PKCδ activation has been shown to enhance NADPH oxidase activity and the levels of reactive oxygen species (ROS) [36]. To investigate if enhanced activation (phosphorylation at Tyr^311^) of PKCδ in VSMC from SHR is implicated in enhanced oxidative stress that has been shown to result in enhanced expression of Gqα and PLCβ1 proteins and protein synthesis [29], the effect of rottlerin on production of O_2_^-^ and NADPH oxidase activity was examined in VSMC from SHR and WKY rats. Results shown in Fig 5, indicate that as reported earlier [33], O_2_^-^ production (A) and NADPH oxidase activity (B) were enhanced in VSMC from SHR as compared to WKY rats by about 110% and 100%, respectively and rottlerin attenuated the enhanced levels of O_2_^-^ as well as NADPH oxidase activity in a concentration-dependant manner and at 10 μM these were completely attenuated to control WKY levels. In addition, rottlerin also attenuated the basal O_2_^-^ and NADPH oxidase activity in VSMC from WKY rats.

### Implication of PKCδ in c-Src activation in VSMC from SHR

**Fig 6:**
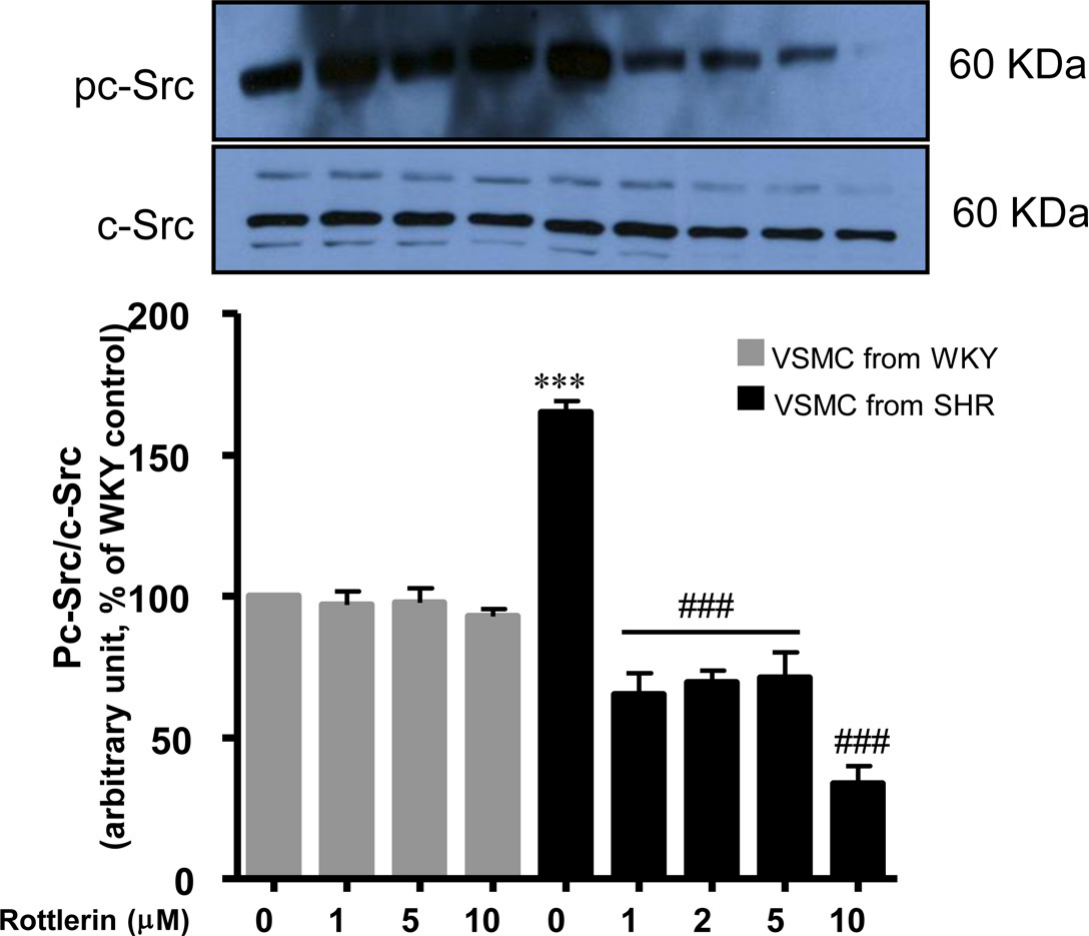
Effect of PKCδ inhibition on c-Src activity in vascular smooth muscle cells (VSMC) from 16-weeks old SHR and age-matched WKY rats. VSMC were incubated in the presence or absence of rottlerin (from 1μM to 10μM) for 16 hours. Membranes were prepared and subjected to Western blotting using specific antibodies (phospho)-c-Src and c-Src (upper panels) as described in Materials and Methods. c-Src phosphorylation level was normalized by total c-Src. The proteins were quantified by densitometric scanning (lower panels). The results are expressed as a percentage of control taken as 100%. Values are means ± SEM of 3 separate experiments using different cell cultures. ***P < 0.001 vs. WKY rats; ### P < 0.001 vs. SHR.

Since PKCδ is implicated in enhanced production of ROS which through c-Src activation was shown to enhance the expression of Gqα and PLCβ1 protein and VSMC hypertrophy in SHR [29], it was of interest to investigate the role of PKCδ in c-Src activation. To test this, the effect of rottlerin on c-Src phosphorylation was examined in VSMC from SHR and WKY rats and the results are shown in Fig 6. The increased phosphorylation of c-Src at Tyr^419^ (70%) was completely attenuated by rottlerin at all the concentrations used (1μM- 5μM) and at 10 μM it was completely abolished whereas it did not have any significant effect in VSMC from WKY rats. Furthermore, the expression level of total c-Src was not altered in VSMC from SHR as compared to WKY rats, and rottlerin did not affect the expression level of c-Src.

### Implication of PKCδ in ERK1/2 Phosphorylation

**Fig 7:**
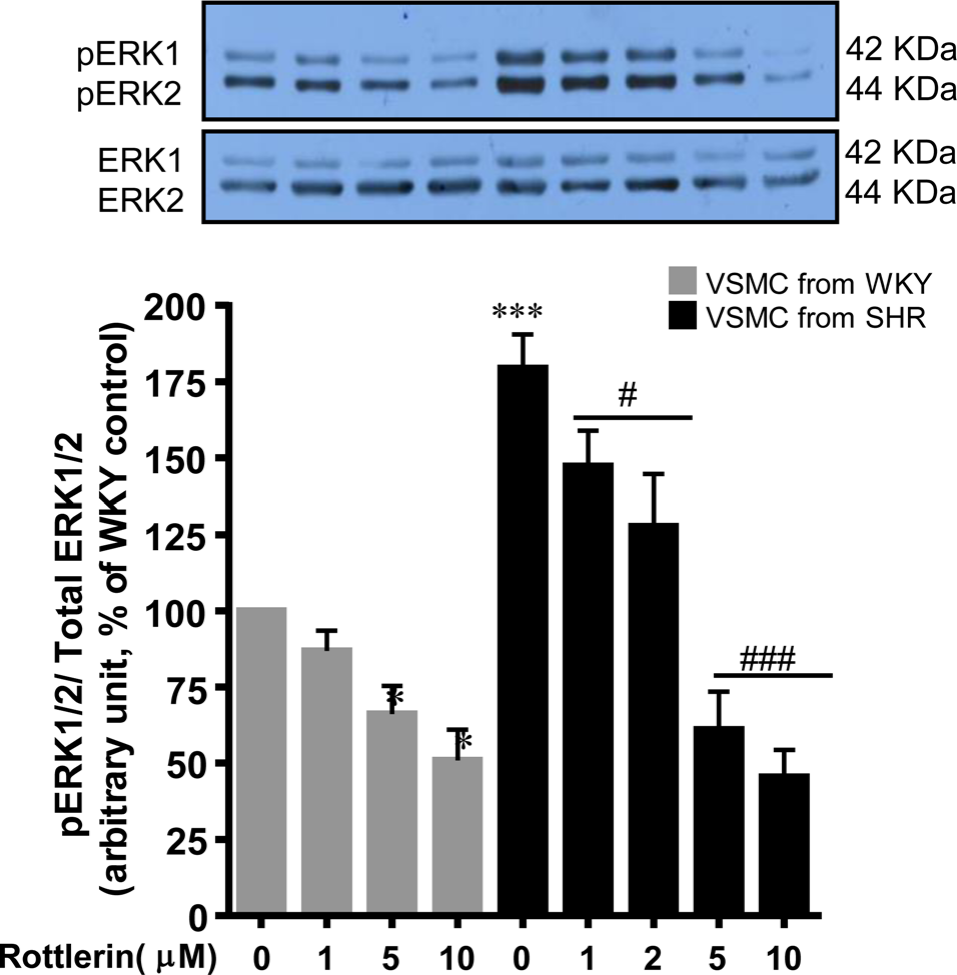
Effect of PKCδ inhibition on p42/44MAPK signaling in vascular smooth muscle cells (VSMC) from 16-weeks old SHR and age-matched WKY rats. VSMC were incubated in the presence or absence of rottlerin (from 1μM to 10μM) for 16 hours. Membranes were prepared and subjected to Western blotting using specific antibodies against pERK1/2 and ERK1/2 (upper panels) as described in Materials and Methods. ERK1/2 phosphorylation level was normalized by total ERK1/2. The proteins were quantified by densitometric scanning (lower panels). The results are expressed as a percentage of control taken as 100%. Values are means ± SEM of 3 separate experiments using different cell cultures. *P < 0.05, ***P < 0.001 vs. WKY rats; # P < 0.05, ### P < 0.001 vs. SHR.

Since enhanced phosphorylation of ERK1/2 was shown to be implicated in enhanced expression of Gqα/PLCβ1 proteins and protein synthesis in VSMC from SHR [2], it was of interest to examine the contribution of PKCδ, an upstream signaling molecule, in the enhanced phosphorylation of ERK1/2 in VSMC from SHR. To test this, we examined the effects of rottlerin on ERK1/2 phosphorylation in VSMC from SHR and WKY rats and the results are shown in Fig 7. As reported earlier [2], ERK1/2 phosphorylation was significantly enhanced by about 75% in VSMC from SHR as compared to WKY rats. Treatment of cells with rottlerin attenuated significantly the enhanced phosphorylation of ERK1/2 in a concentration-dependant manner in VSMC from SHR and at 5 and 10 μM, it was completely attenuated to control level. In addition, rottlerin at 5 μM and 10 μM inhibited significantly the ERK1/2 phosphorylation in VSMC from WKY rats by about 35% and 50% respectively.

### Implication of PKCδ in growth factor receptor expression and activation

**Fig 8:**
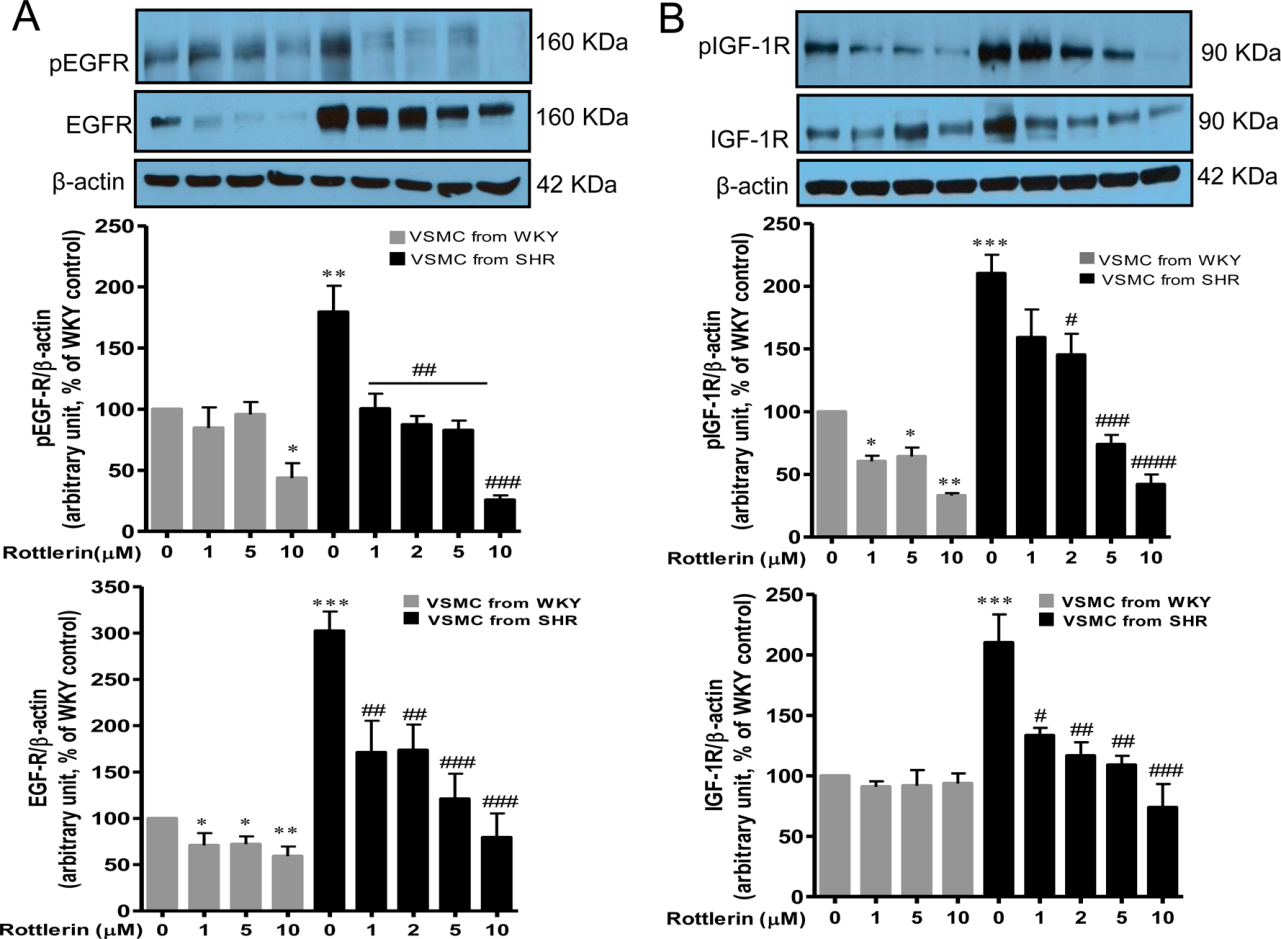
Effect of rottlerin on EGF-R and IGF-1R phosphorylation and expression in vascular smooth muscle cells (VSMC) from 16-weeks old SHR and age-matched WKY rats. VSMC were treated with different concentration of rottlerin for 16 hours. The cell lysates were prepared and subjected to Western blotting using specific antibodies against EGF-R/pEGF-R and IGF-1R/p-IGF-1R (A, B upper panels) as described in Materials and methods. The proteins were quantified by densitometric scanning (A, B lower panels). The results are expressed as percentage of control, taken as 100%. Values are means ± SEM of 5 separate experiments using different cell cultures. * P < 0.05, **P < 0.01, ***P < 0.001 vs. WKY rats; # P < 0.05, ## P < 0.01, ### P < 0.001 vs. SHR.

We earlier showed that growth factor receptor transactivation and MAP Kinase signaling plays a role in the overexpression of Gqα and PLCβ1 proteins and VSMC hypertrophy in VSMC from SHR [29]. To further investigate the role of PKCδ in growth factor receptor transactivation and/or expression, the effect of rottlerin on the phosphorylation of EGFR and IGF-1R using specific phospho-tyrosine antibodies was examined in VSMC from SHR and the results are shown in Fig 8. Phospho-specific-Tyr^1173^-EGFR detected a single band at 160 kDa (A) and phospho-specific-Tyr^1165/1166^-IGF-1R detected a single band at 90 kDa corresponding to IGF-1R (B), in VSMC from both SHR and WKY rats. However, as reported earlier [29], the extent of growth factor receptor phosphorylation was greater in VSMC from SHR compared to VSMC from WKY rats. The phosphorylation of IGF-1R was increased by approximately 110% in VSMC from SHR compared to WKY rats, whereas the phosphorylation of EGFR was augmented by about 80%. The enhanced phosphorylation of EGF-R at Tyr^1173^ was attenuated completely by rottlerin (from 1μM to 5μM), whereas these concentrations of rottlerin were ineffective in reducing significantly the phosphorylation level of EGFR in VSMC from WKY. At a concentration of 10μM, rottlerin completely abolished the enhanced phosphorylation of EGFR in VSMC from SHR and attenuated the phosphorylation level of EGFR by about 50% in VSMC from WKY rats. Rottlerin inhibited significantly the increased phosphorylation of IGF-1R (B) in a concentration dependant manner. Furthermore, these treatments also decreased the phosphorylation level in VSMC from WKY. In addition, the expression levels of EGFR (A) and IGF-1R (B) were also augmented by about 200% and 100% respectively in VSMC from SHR as compared to WKY rats, which was attenuated significantly by rottlerin. Furthermore, rottlerin also attenuated significantly the protein expression of EGFR in VSMC from WKY rats.

## Discussion

Ang II-induced VSMC hypertrophy has been shown to involve PKCδ [7]. In addition, this serine-threonine kinase was also reported to be involved in the processes of cardiac hypertrophy [37,38]. We earlier showed the role of endogenous Ang II and ET-1 in the enhanced expression of Gqα and PLCβ1 proteins and the enhanced protein synthesis in VSMC from SHR through MAPKs singling [2]. In the present study, we demonstrate the implication of enhanced PKCδ activation (associated with PKCδ phosphorylation at Tyr^311^) in the enhanced expression of Gqα and PLCβ1 proteins and enhanced protein synthesis in VSMC form spontaneously hypertensive rats (SHR).

We show that VSMC from 16-week-old SHR exhibit enhanced PKCδ phosphorylation at Tyr^311^ which may contribute to the enhanced expression of Gqα and PLCβ1 proteins as well as enhanced protein synthesis because PKCδ inhibition with rottlerin, as quantified by the phosphorylation level at Tyr^311^, attenuated significantly the enhanced expression of Gqα and PLCβ1 proteins and the enhanced protein synthesis in VSMC from SHR. This was further supported by our study showing that knockdown of PKCδ with specific siRNA attenuated significantly the enhanced protein synthesis in VSMC from SHR. Our results are in agreement with the study of Nakashima et al who have shown the implication of PKCδ associated with enhanced phosphorylation at Tyr^311^ in Ang II-induced VSMC hypertrophy [7]. Furthermore, the fact that rottlerin also inhibits the PKCδ phosphorylation, the expression of Gqα/PLCβ1 as well as protein synthesis in control cells, suggests the implication of endogenous PKCδ in eliciting these responses. It should be noted that rottlerin has been used as a putative inhibitor of PKCδ in several studies during the last 20 years in order to block the activity of PKCδ [39,40], that was correlated with increased PKCδ phosphorylation [41]. In addition, rottlerin was also shown to inhibit PKCδ activity through mitochondria uncoupling mechanism [42].

PKCδ has been shown to be activated by hyperglycemia-induced oxidative stress [43]. Furthermore, PKCδ activation was also associated with the process of atherosclerosis implicating LDL oxidation [44]. Our results showing that the inhibition of PKCδ activity with rottlerin results in a significant attenuation of NADPH oxidase activity and O_2_^-^ production and suggests the implication of PKCδ in ROS production in VSMC. Our results are consistent with other studies demonstrating the inhibition of ROS production by rottlerin in many cell types including VSMCs and adipocytes [45,46]. A role of PKCδ in NADPH oxidase activation has been reported in various cells [47]. The mechanisms by which PKCδ activates NADPH oxidase is not clear but may involve the phosphorylation of the cytoplasmic subunits of the oxidase, such as p47*phox*, and initiates its translocation to the membrane [48,49]. In this regard, a role of PKCδ in p47^phox^ activation of NADPH oxidase in Ang II–induced ROS production and VSMC hypertrophy has been shown [36]. In addition, PKCδ activation has also been shown to mediate its effects through increasing ROS production and NADPH subunits expression [26, 50]. We earlier showed that enhanced oxidative stress exhibited by VSMC from SHR contributes to the overexpression of Gqα and PLCβ1 proteins as well as to enhanced protein synthesis [29]. Taken together, it may be suggested that PKCδ-induced enhanced expression of Gqα and PLCβ1 proteins as well as enhanced protein synthesis in VSMC from SHR may also be mediated through its ability to increase oxidative stress.

We earlier showed a role of c-Src in enhanced expression of Gqα and PLCβ1 proteins as well as in enhanced protein synthesis in VSMC from SHR [29]. In this study, we show that the augmented phosphorylation of c-Src in VSMC from SHR is attributed to the enhanced activation of PKCδ because the inhibition of PKCδ by rottlerin attenuated the enhanced phosphorylation of c-Src. Furthermore, the oxidative stress has been demonstrated to be the upstream signaling molecule of c-Src [2]. Taken together, it may be suggested that the enhanced activation of PKCδ through oxidative stress and activation of c-Src contributes to the overexpression of Gqα and PLCβ1 proteins as well as to the enhanced protein synthesis in VSMC from SHR.

As demonstrated in this study, VSMC from SHR exhibit enhanced expression of EGFR and IGF-1R which may be associated with cell dedifferentiation to a synthetic profile characterized by enhanced protein synthesis. The implication of growth factor receptors in enhanced expression of Gqα and PLCβ1 proteins and enhanced protein synthesis in VSMC from SHR has been shown in an earlier study[29]. These results suggest that the enhanced protein expression of Gqα/PLCβ1 as well as EGFR and IGF-1R may reflect a phenotype switch of VSMC from a contractile state to a synthetic state in SHR. In fact, VSMC profile conversion is associated with the modulation of protein expression of certain membrane molecules implicated in VSMC hypertrophy, proliferation and contractility [51,52]. In addition, we also showed that c-Src is the upstream of growth factor receptor activation [29]. The transactivation of several receptor tyrosine kinases (RTKs) has also been reported to involve c-Src [31,53,54]. Our results showing that rottlerin inhibited the increased phosphorylation of IGF-1R and EGFR, suggest a role of PKCδ in enhanced activation of growth factor receptors in VSMC from SHR. In this regard, Hsieh et al have shown a pivotal role of PKCδ in thrombin- induced EGFR expression in VSMC [8].

A role of MAPK in enhanced expression of Gqα/PLCβ1 proteins and enhanced protein synthesis in VSMC from SHR has been shown [2]. Growth factor receptors have also been reported to signal through MAP kinase pathways [31,55]. The fact that rottlerin inhibited the enhanced phosphorylation of ERK1/2 in VSMC from SHR suggests the implication of PKCδ in enhanced activation of MAP kinase. In this context, the inhibition of PKCδ with rottlerin has been reported to inhibit the ET-1- induced MAPK activity in VSMC [56]. Taken together, it may be suggested that enhanced activity of PKCδ in VSMC from SHR through the transactivation of growth factor receptors and MAP kinase signaling increase the expression of Gqα and PLCβ1 and results in VSMC hypertrophy.

**Fig 9:**
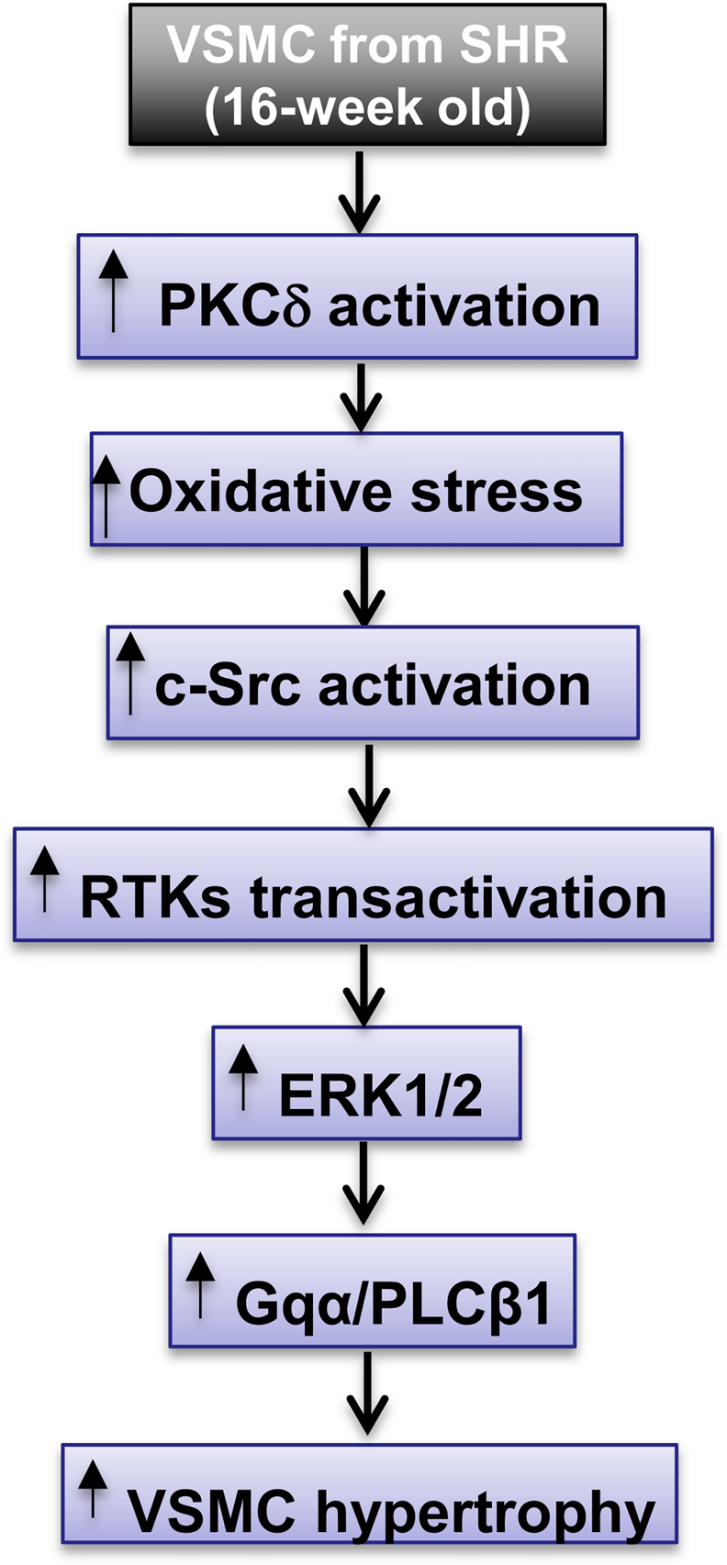
The possible intracellular signaling mechanisms implicated in PKCδ-induced enhanced expression of Gqα/PLC-β1 proteins and VSMC hypertrophy in SHR. PKCδ through the activation of ROS production and c-Src, trans-activate RTKs and MAPK that increases the protein expression of Gqα/PLC-β1 and results in enhanced protein synthesis.

In summary, we provide evidence that the enhanced activation of PKCδ at Tyr^311^ phosphorylation in VSMC from SHR through oxidative stress and c-Src, transactivate growth factor receptor which increases the activity of MAP kinase and contributes to the enhanced expression of Gqα and PLCβ1 proteins and VSMC hypertrophy (Fig 9). In addition, we also show for the first time that rottlerin that has been shown to exert neuro-protective effect in both cell culture and preclinical animal models of Parkinson disease [57], anticancer effects in a variety of tumor cell types [58] and induces apoptosis in chronic lymphocytic leukaemia (CLL) cells [59] also inhibits VSMC hypertrophy in SHR model of hypertension. From these studies, it may be suggested that PKCδ protein may serve as a potential target for the development of new therapies for the treatment of cardiovascular diseases.
